# Why do endocrine profiles in elite athletes differ between sports?

**DOI:** 10.1186/s40842-017-0050-3

**Published:** 2018-02-07

**Authors:** Peter H. Sönksen, Richard I. G. Holt, Walailuck Böhning, Nishan Guha, David A. Cowan, Christiaan Bartlett, Dankmar Böhning

**Affiliations:** 1 Human Development and Health Academic Unit, University of Southampton Faculty of Medicine, Southampton, UK; 2 Southampton Statistical Sciences Research Institute, University of Southampton, Southampton, UK; 3 Nuffield Division of Clinical Laboratory Sciences, University of Oxford, Oxford, UK; 4 Department of Pharmacy and Forensic Science, Drug Control Centre, King’s College London, London, UK

**Keywords:** Elite-sport, Endocrine-profiles, Body-composition, BMI, Collagen biomarkers

## Abstract

**Background:**

Endocrine profiles have been measured on blood samples obtained immediately post-competition from 693 elite athletes from 15 Olympic Sports competing at National or International level; four were subsequently excluded leaving 689 for the current analysis.

**Methods:**

Body composition was measured by bioimpedance in a sub-set of 234 (146 men and 88 women) and from these data a regression model was constructed that enabled ‘estimated’ lean body mass and fat mass to be calculated on all athletes. One way ANOVA was used to assess the differences in body composition and endocrine profiles between the sports and binary logistical regression to ascertain the characteristic of a given sport compared to the others.

**Results:**

The results confirmed many suppositions such as basketball players being tall, weightlifters short and cross-country skiers light. The hormone profiles were more surprising with remarkably low testosterone and free T3 (tri-iodothyronine) in male powerlifters and high oestradiol, SHBG (sex hormone binding globulin) and prolactin in male track and field athletes. Low testosterone concentrations were seen 25.4% of male elite competitors in 12 of the 15 sports and high testosterone concentrations in 4.8% of female elite athletes in 3 of the 8 sports tested. Interpretation of the results is more difficult; some of the differences between sports are at least partially due to differences in age of the athletes but the apparent differences between sports remain significant after adjusting for age. The prevalence of ‘hyperandrogenism’ (as defined by the IAAF (International Association of Athletics Federations) and IOC (International Olympic Committee)) amongst this cohort of 231 elite female athletes was the highest so far recorded and the very high prevalence of ‘hypoandrogenism’ in elite male athletes a new finding.

**Conclusions:**

It is unclear whether the differences in hormone profiles between sports is a reason why they become elite athletes in that sport or is a consequence of the arduous processes involved. For components of body composition we know that most have a major genetic component and this may well be true for endocrine profiles.

**Electronic supplementary material:**

The online version of this article (10.1186/s40842-017-0050-3) contains supplementary material, which is available to authorized users.

## Background

The first report on endocrine hormone profiles was in a group of 693 elite athletes across a range of Olympic Sports in 2014 [1]. In addition to statistically significant differences in profiles between men and women, there were considerable differences between athletes from various sports.

It has long been known that different sports attract athletes who differ in body composition; for example, marathon runners and cross-country skiers are thin and light while weightlifters and powerlifters are short and stocky and basketball players tall. Healy et al. also showed that on average elite female athletes had a lean body mass (LBM) that was 85% of the LBM of elite male athletes and proposed that the differences in strength and world records between men and women reflected this [1]. There is no clear indication why men and women develop bodies that show a fundamental difference in lean and fat mass but it is possible, if not likely, that it is partly due to differences in hormonal profiles between the sexes [2].

There are very limited published data on endocrine profiles in sport, most being confined to a single sport. One unexpected finding of Healy et al. [1] was that 16.5% of male elite athletes had testosterone concentrations less than the lower limit of the laboratory reference range for ‘normal’ men and that 13.7% of elite female athletes had a testosterone concentration greater than the laboratory reference interval for ‘normal’ women including several with values within the reference range for men.

This paper examines these differences in endocrine profiles discovered by Healy et al. in more detail and attempts to interpret some of the findings.

## Methods

### Participants

The details of recruitment of the volunteer elite athletes as part of the GH-2000 study (A Methodology for the Detection of Doping with Growth Hormone & Related Substances. EU Contract Number: BMH4 CT950678) and the subsequent collection of data including analysis of blood samples have been published previously [3] as has the selection of the sub-set of these athletes in whom endocrine profiles were measured [1]. The participants in this study are those previously published. In brief, athletes were recruited on an ‘opportunistic’ basis from the 15 Olympic sports that were interested and prepared to co-operate with the GH-2000 research project whose aim was to develop a test to detect growth hormone misuse in professional athletes. Samples were collected within two hours of completion of their event. The project was funded mainly by the European Union and International Olympic Committee with further support from the industries and universities involved. Volunteers gave written consent to participation and this included a statement confirming that they had not misused any banned drug or anabolic agent and this was confirmed by finding no abnormal testosterone/LH ratios. Results of endocrine profiles were available in 694 of the original cohort of 813 elite athletes recruited for the original GH-2000 ‘Cross-Sectional’ study [3]; they were those individuals with sufficient serum left for analysis of an endocrine profile after completion of the main study. Three participants were excluded as there was only 1 volunteer from each sport (women Powerlifting, Marathon and Canoeing) and one man was excluded as his thyroid profile showed him to be markedly hyperthyroid (high fT3 and suppressed TSH (thyroid stimulating hormone)) leaving 689 individuals for the current analysis.

### Ethics approval

All volunteers gave written informed consent to participate in the original study including subsequent analysis and publication of the data. The study was approved by the Ethics Committee of West Lambeth Health Authority (as the committee covering the co-ordinating centre St Thomas’ Hospital, London) and the appropriate local ethics committees of all participating partners.

### Body composition

**Fig. 1:**
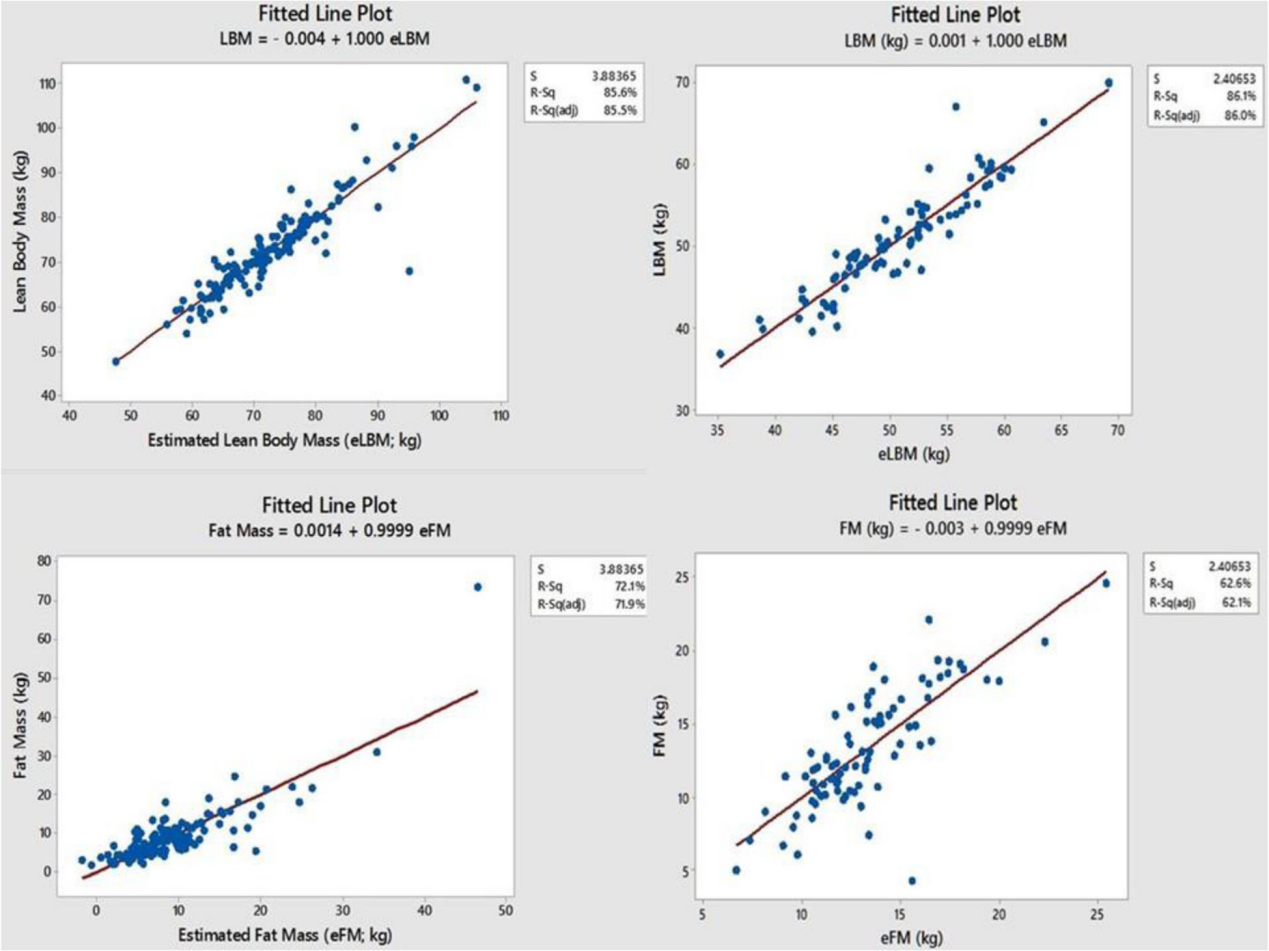
Lean body mass (LBM) and Fat Mass (FM) measured by bio-impedance are compared with the same variables estimated from just height and weight. There were 146 men and 88 women, R-squared values of 86, 85.5, 71.9 and 62.1% show the model to be reasonably accurate (R^2^ = Percentage of response variable variation that is explained by its relationship with one or more predictor variables. In general, the higher the R^2^, the better the model fits your data. R^2^ is always between 0 and 100%. It is also known as the coefficient of determination or multiple determination (in multiple regression). The adjusted R-squared is a modified version of R-squared that has been adjusted for the number of predictors in the model. S represents the standard deviation of the distance between the data values and the fitted values

Demographic data included self-reported height, weight and age. Weight and body composition was measured on a sub-set of 234 (146 men and 88 women) at events where it was possible to use the Tanita TB7–305 bioimpedance analyser; this was only swimming, rowing and track and field. Since the measured body composition data were only available for three sports, estimated lean body mass (eLBM) and estimated fat mass (eFM) were calculated for everyone using regression equations (for each sex, using height and weight) derived from those in the three sports in whom body composition was measured; these data are shown in Fig. 1. Estimated fat mass was calculated by subtracting eLBM from total body weight. The large R^2^ values and slope of almost unity indicate that the statistical models have a reasonable degree of validity.

$$ \mathrm{For}\ \mathrm{men}:\kern0.5em \mathrm{eLBM}=\hbox{-} 43.68+0.4598\ \mathrm{weight}+0.4285\ \mathrm{height}\ \left(\mathrm{N}=146;{\mathrm{R}}^2=85.6\%\right) $$

$$ \mathrm{For}\ \mathrm{women}:\kern0.5em \mathrm{eLBM}=\hbox{-} 22.68+0.5157\ \mathrm{weight}+0.2354\ \mathrm{height}\ \left(\mathrm{N}=88;{\mathrm{R}}^2=86.1\%\right) $$

### Endocrine measurements

The endocrine profiles and the methods used are detailed in Healy et al. [1] and [3]. Serum growth hormone (GH), IGF-I (Insulin-like growth factor 1), pro-collagen type III N-terminal peptide (P-III-NP), carboxy-terminal cross-linked telopeptide of type I collagen (ICTP), carboxy-terminal propeptide of type I collagen (PICP) and osteocalcin were determined at the Sahlgrenska Hospital (Gothenburg, Sweden) and IGFBP-2 (IGF Binding Protein #2), IGFBP-3 (IGF Binding Protein #3) and acid-labile subunit (ALS) were measured at the Kolling Institute (Sydney, Australia). Serum luteinising hormone (LH), follicle-stimulating hormone (FSH), prolactin, thyroid-stimulating hormone (TSH), free tri-iodothyronine (fT3), free thyroxine (fT4), oestradiol, cortisol, testosterone and sex-hormone binding globulin (SHBG) were measured in the endocrine laboratory at St Thomas’ Hospital (London, UK) using the Siemens Centaur and Immulite platforms. Many of the oestradiol concentrations (160/644 men and 64/234 women) were less than the laboratory Lower Limit of Quantification of 34 pmol/l and in the statistical analyses these were treated as missing data. The lower part of the female range of testosterone was initially established with a radio-immunoassay (RIA) and then the RIA was correlated with the automated method. The automated assay had a between-assay imprecision of 20% at 1.5 nmol/l.

### Statistical analysis

All analyses were performed using Minitab 17 with a significance level set at 0.05 unless stated to the contrary.

Comparison of concentration of a given hormone or marker between sports was by one-way analysis of variance. Comparison between the sport with the lowest mean value of a given endocrine variable and the results from other sports was performed using Dunnett’s method (Minitab 17).

On occasions where a given variable was known to be age-dependent (e.g. growth hormone, IGF-I and the collagen biomarkers), multiple regression analysis with sport and age as independent variables, was used to examine their relative contributions to the observed differences. Outliers were included in the data for analysis and not treated separately; however, in these cases analysis was repeated after log-transformation of the data and no occasion did this affect the outcome.

Using Binary Logistic Regression the demographic data and endocrine profiles of a given sport was compared with that of the ‘control’ group created from all the other sports combined. This approach allowed the determination of the endocrine variables that were statistically characteristic in a given sporting group (either positively or negatively associated with that sport).

There are in all 390 comparisons so one would expect 20 to be positive by chance alone at the *p* < 0.05 level (in fact 21 men and 12 women), 4 at the *p* < 0.01 level (21 men and 7 women) and 1 at the level of *p* < 0.001 (39 men and 16 women)). Thus only variables with an association at the 1/100 (p < 0.01) or better were accepted as relevant.

## Results

**Fig. 2:**
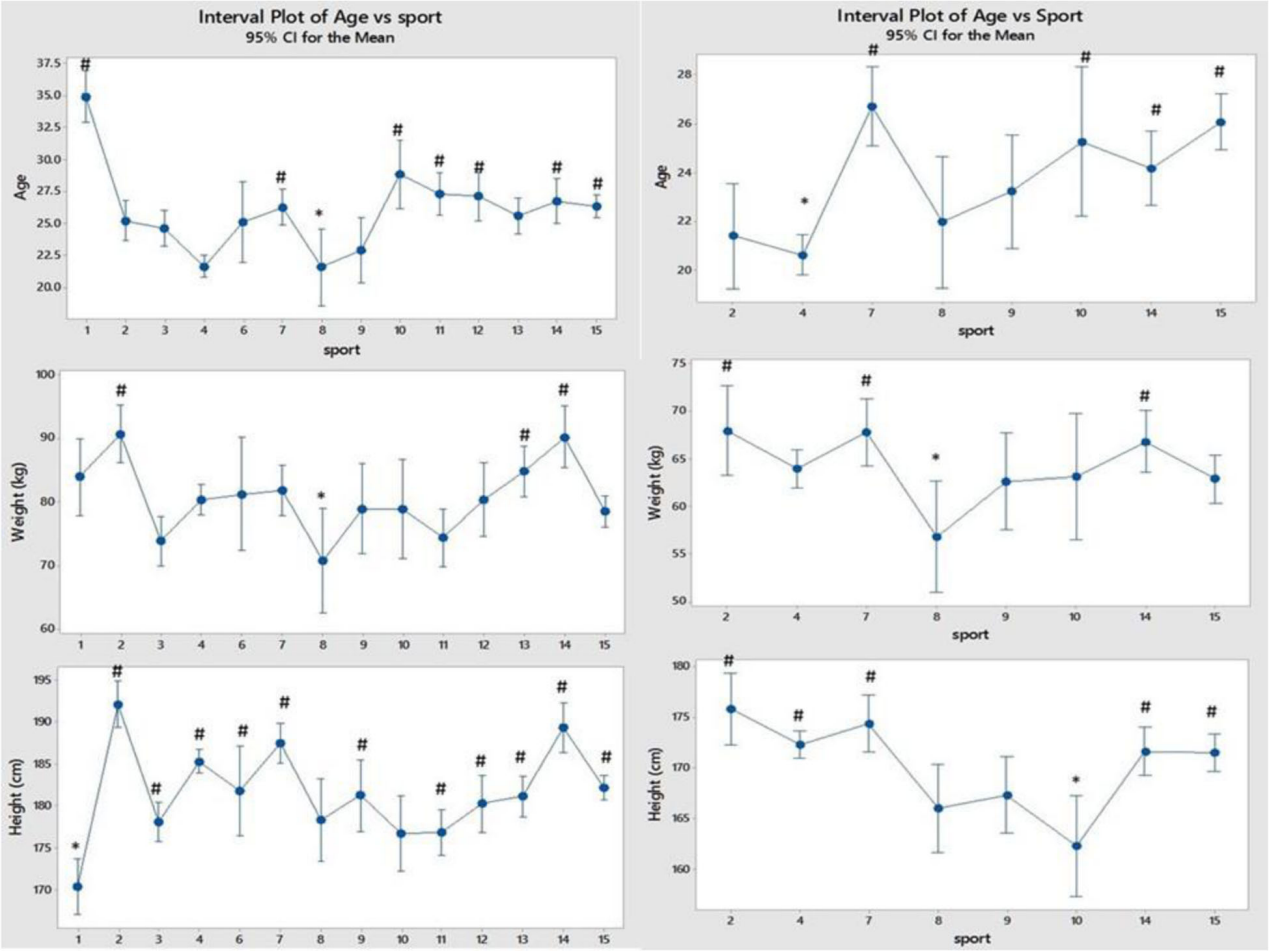
The differences in mean age, weight and height between sports. For all the figures data from men are shown in the left panel and those from women are in the right panel. The lowest mean for each variable is marked with * and means that are significantly higher than this by one-way analysis of variance are marked with #. Each sport is represented by a numerical code and M = men and W = women: 1-Power Lifting (18 M and 1 W), 2-Basketball (27 M and 14 W), 3-Football (Soccer; 37 M), 4 Swimming (100 M and 91 W), 5-Marathon (1 W), 6-Canoeing (7 M and 1 W), 7-Rowing (36 M and 25 W), 8-Cross Country Skiing (8 m and 9 W), 9-Alpine Skiing (11 M and 12 W), 10-Weight Lifting (10 M and 7 W), 11-Judo (26 M), 12-Bandy (19 M), 13-Ice Hockey (38 M), 14 Handball (23 M and 29 W) and 15-Track and Field (95 M and 49 W)

**Fig. 3:**
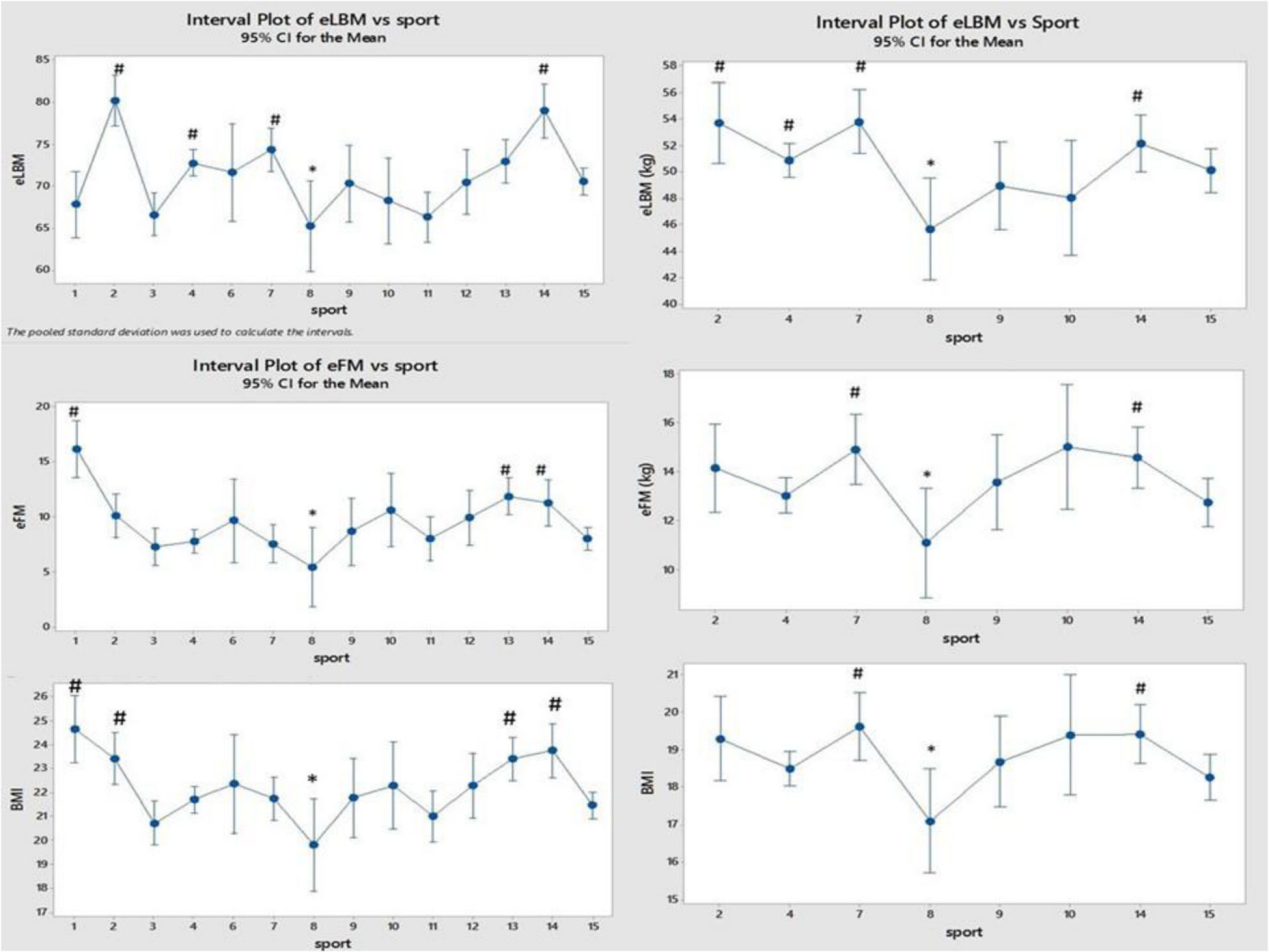
Comparison of eLBM, eFM and BMI between the sports and between the sexes (men on the left and women on the right). Each sport is represented by a numerical code and M = men and W = women: 1-Power Lifting (18 M and 1 W), 2-Basketball (27 M and 14 W), 3-Football (Soccer; 37 M), 4 Swimming (100 M and 91 W), 5-Marathon (1 W), 6-Canoeing (7 M and 1 W), 7-Rowing (36 M and 25 W), 8-Cross Country Skiing (8 m and 9 W), 9-Alpine Skiing (11 M and 12 W), 10-Weight Lifting (10 M and 7 W), 11-Judo (26 M), 12-Bandy (19 M), 13-Ice Hockey (38 M), 14 Handball (23 M and 29 W) and 15-Track and Field (95 M and 49 W)

**Fig. 4:**
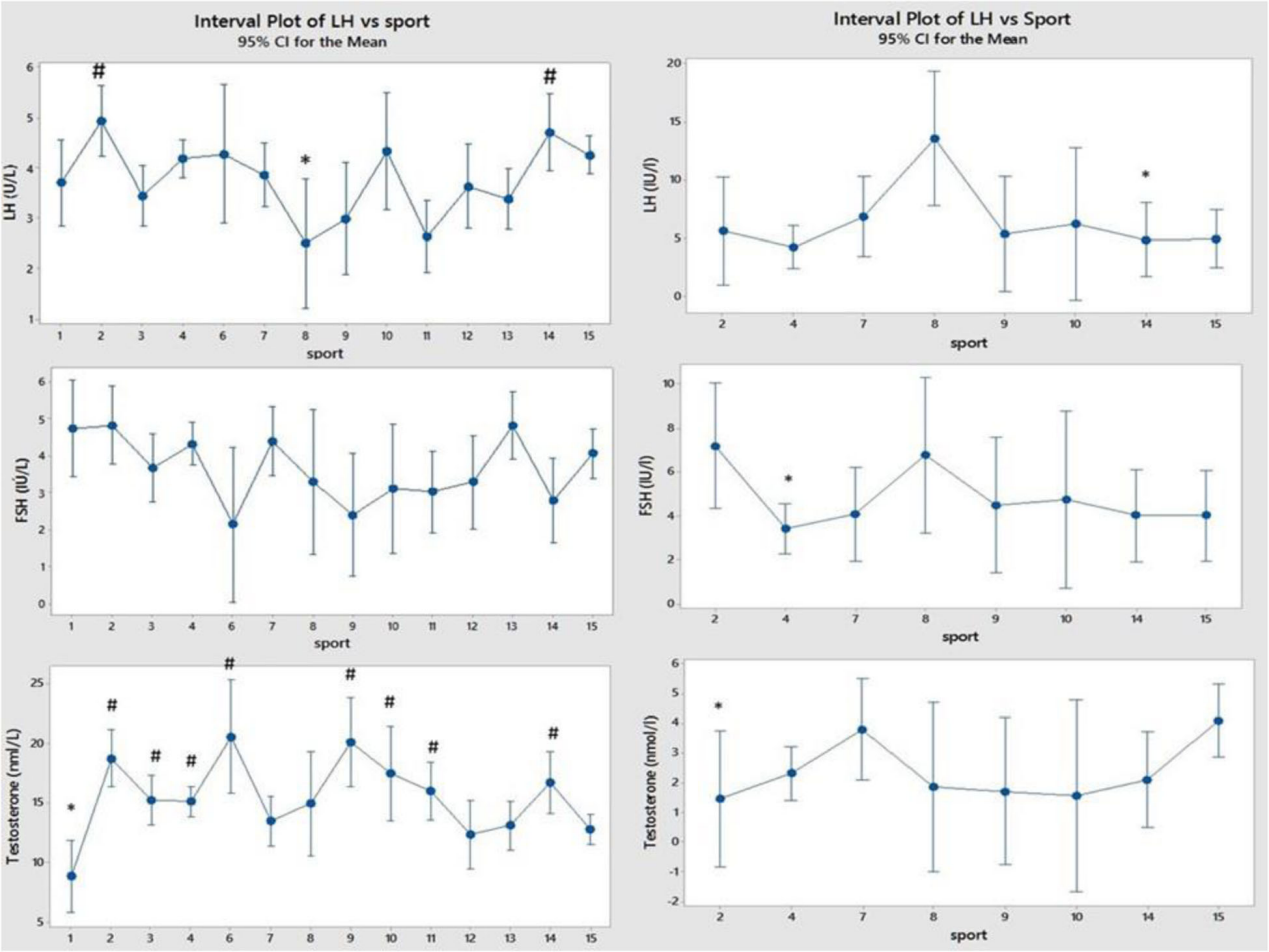
Comparison of LH, FSH and Testosterone between sports and between sexes (men on the left and women on the right). Each sport is represented by a numerical code and M = men and W = women: 1-Power Lifting (18 M and 1 W), 2-Basketball (27 M and 14 W), 3-Football (Soccer; 37 M), 4 Swimming (100 M and 91 W), 5-Marathon (1 W), 6-Canoeing (7 M and 1 W), 7-Rowing (36 M and 25 W), 8-Cross Country Skiing (8 m and 9 W), 9-Alpine Skiing (11 M and 12 W), 10-Weight Lifting (10 M and 7 W), 11-Judo (26 M), 12-Bandy (19 M), 13-Ice Hockey (38 M), 14 Handball (23 M and 29 W) and 15-Track and Field (95 M and 49 W)

**Fig. 5:**
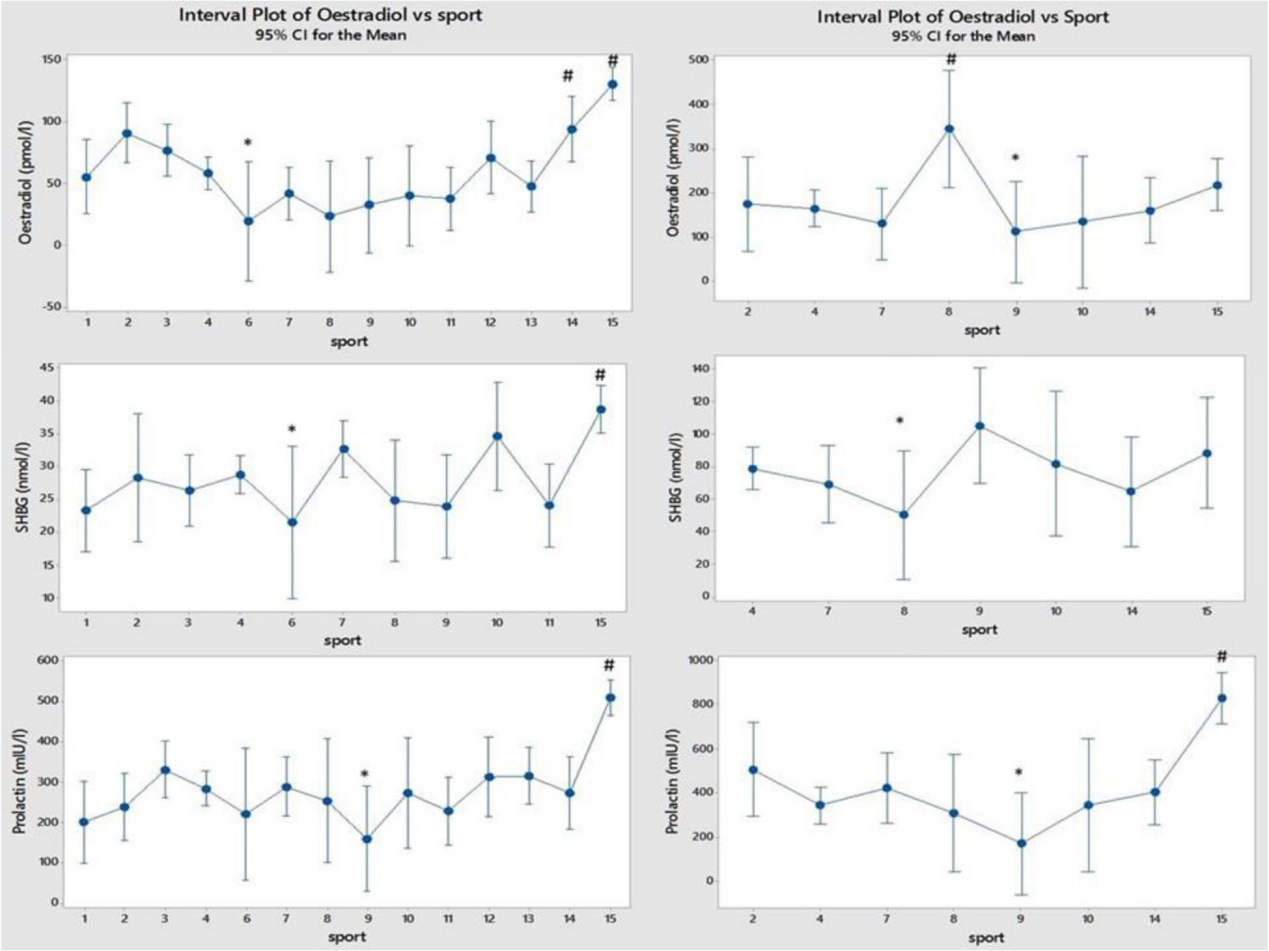
Comparison of Oestradiol, Sex Hormone Binding Globulin (SHBG) and Prolactin between sports, (men on the left and women on the right). Each sport is represented by a numerical code and M = men and W = women: 1-Power Lifting (18 M and 1 W), 2-Basketball (27 M and 14 W), 3-Football (Soccer; 37 M), 4 Swimming (100 M and 91 W), 5-Marathon (1 W), 6-Canoeing (7 M and 1 W), 7-Rowing (36 M and 25 W), 8-Cross Country Skiing (8 m and 9 W), 9-Alpine Skiing (11 M and 12 W), 10-Weight Lifting (10 M and 7 W), 11-Judo (26 M), 12-Bandy (19 M), 13-Ice Hockey (38 M), 14 Handball (23 M and 29 W) and 15-Track and Field (95 M and 49 W)

**Fig. 6:**
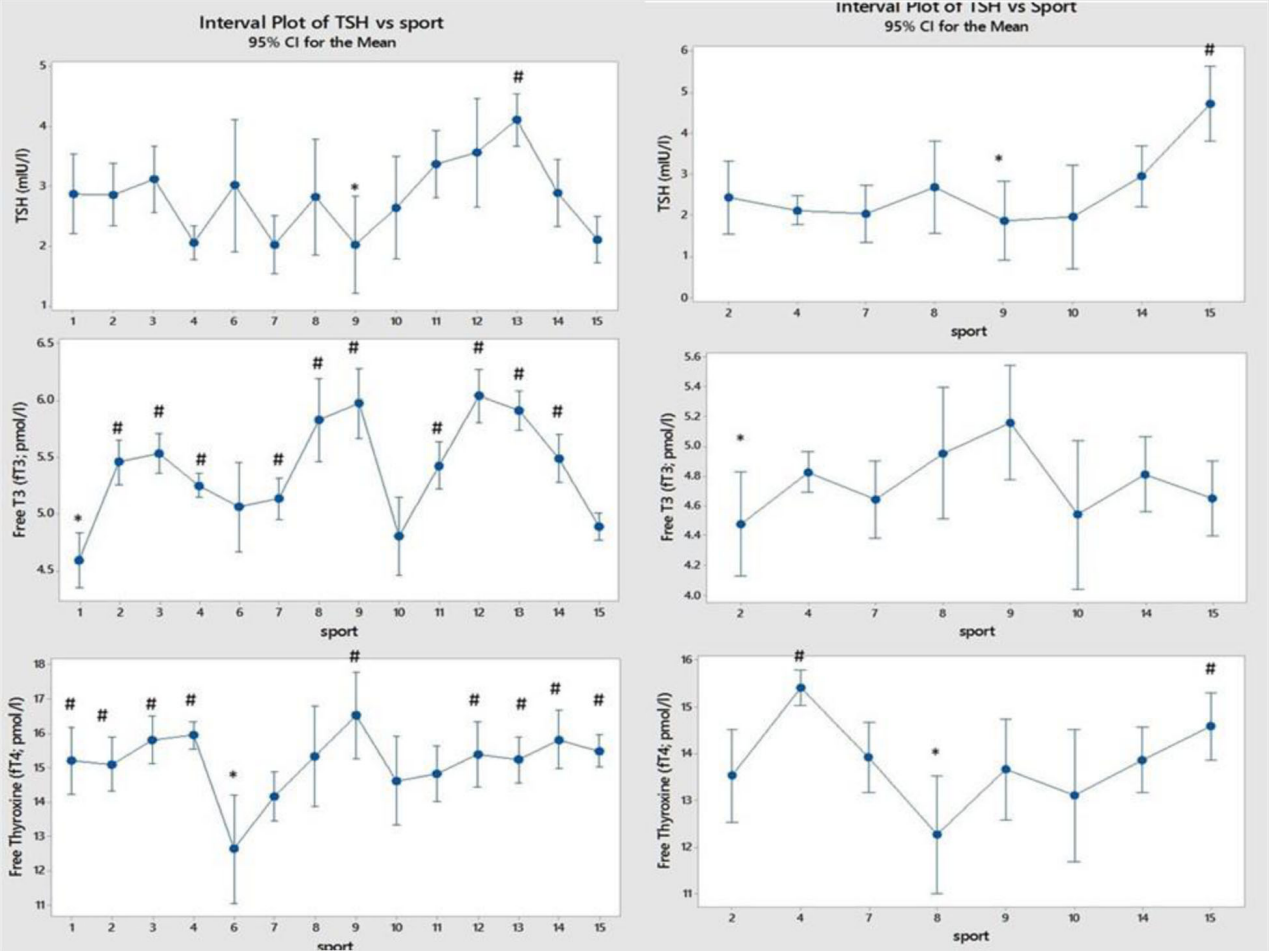
Comparison of thyroid function test results between sports (men on the left and women on the right). Each sport is represented by a numerical code and M = men and W = women: 1-Power Lifting (18 M and 1 W), 2-Basketball (27 M and 14 W), 3-Football (Soccer; 37 M), 4 Swimming (100 M and 91 W), 5-Marathon (1 W), 6-Canoeing (7 M and 1 W), 7-Rowing (36 M and 25 W), 8-Cross Country Skiing (8 m and 9 W), 9-Alpine Skiing (11 M and 12 W), 10-Weight Lifting (10 M and 7 W), 11-Judo (26 M), 12-Bandy (19 M), 13-Ice Hockey (38 M), 14 Handball (23 M and 29 W) and 15-Track and Field (95 M and 49 W)

**Fig. 7:**
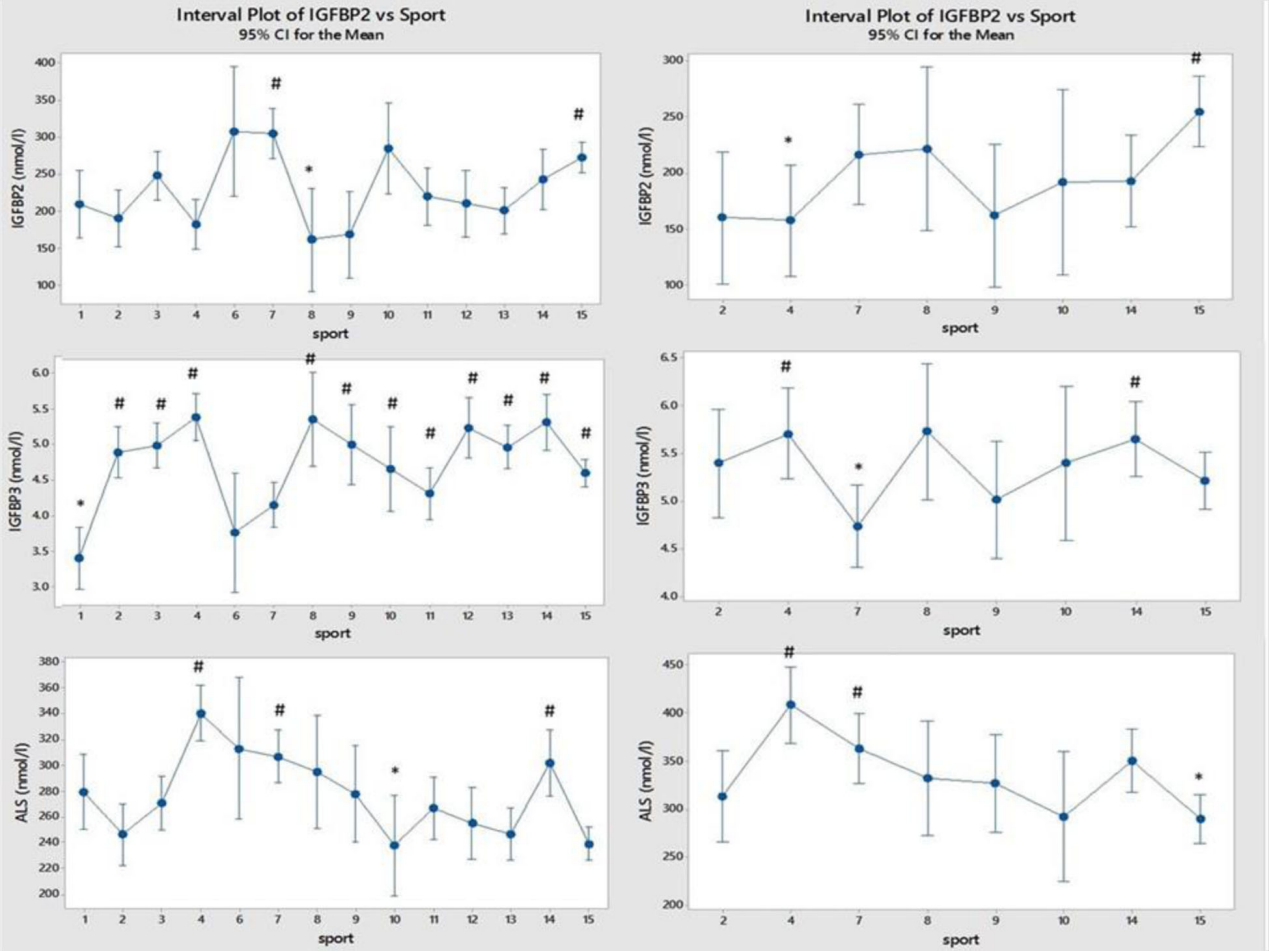
Comparison of IGF binding-protein concentrations between sports (men on the left and women on the right). Each sport is represented by a numerical code and M = men and W = women: 1-Power Lifting (18 M and 1 W), 2-Basketball (27 M and 14 W), 3-Football (Soccer; 37 M), 4 Swimming (100 M and 91 W), 5-Marathon (1 W), 6-Canoeing (7 M and 1 W), 7-Rowing (36 M and 25 W), 8-Cross Country Skiing (8 m and 9 W), 9-Alpine Skiing (11 M and 12 W), 10-Weight Lifting (10 M and 7 W), 11-Judo (26 M), 12-Bandy (19 M), 13-Ice Hockey (38 M), 14 Handball (23 M and 29 W) and 15-Track and Field (95 M and 49 W)

**Fig. 8:**
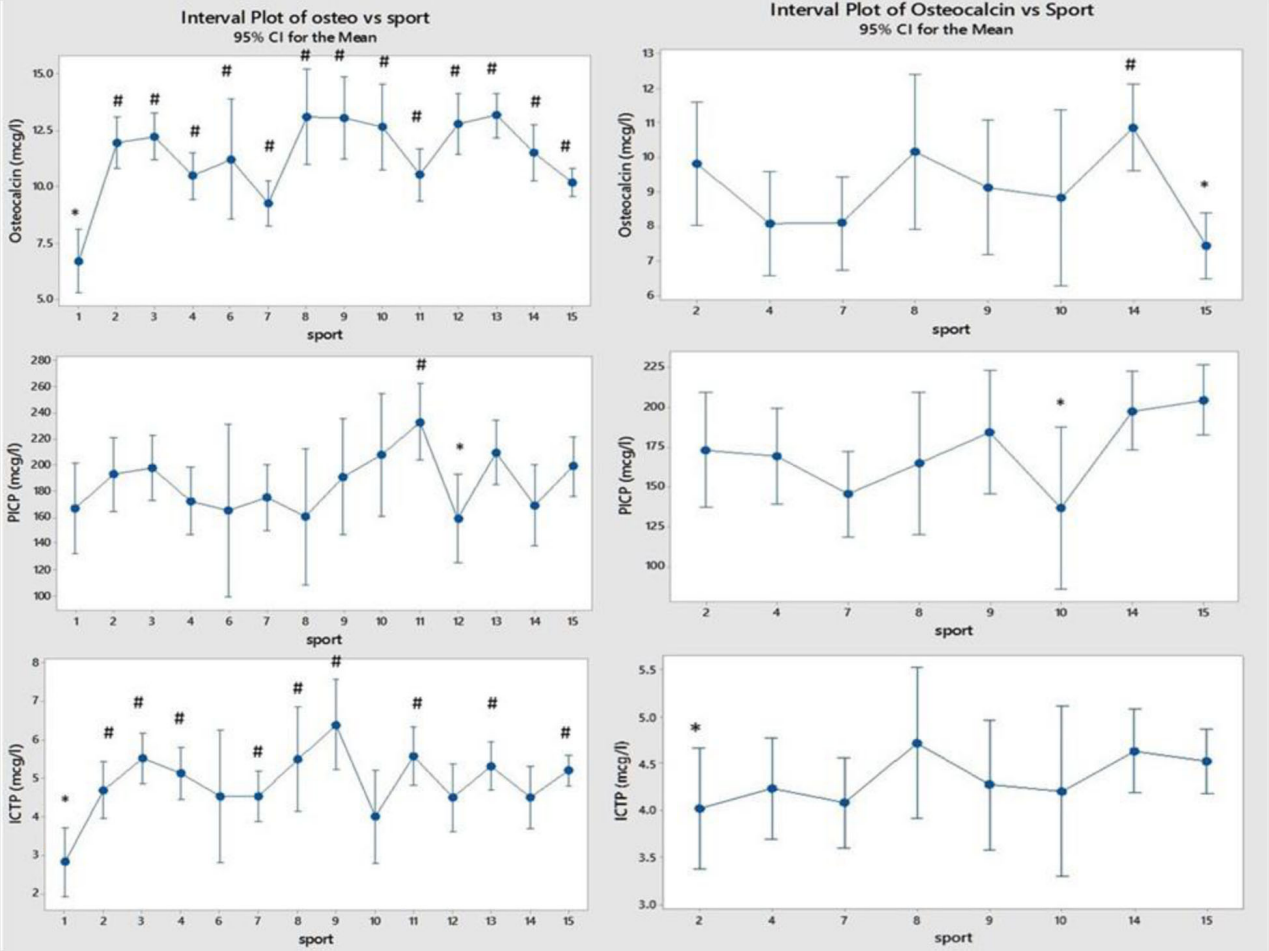
Comparison of Osteocalcin and the collagen peptides PICP and ICTP between sports (men on the left and women on the right). Each sport is represented by a numerical code and M = men and W = women: 1-Power Lifting (18 M and 1 W), 2-Basketball (27 M and 14 W), 3-Football (Soccer; 37 M), 4 Swimming (100 M and 91 W), 5-Marathon (1 W), 6-Canoeing (7 M and 1 W), 7-Rowing (36 M and 25 W), 8-Cross Country Skiing (8 m and 9 W), 9-Alpine Skiing (11 M and 12 W), 10-Weight Lifting (10 M and 7 W), 11-Judo (26 M), 12-Bandy (19 M), 13-Ice Hockey (38 M), 14 Handball (23 M and 29 W) and 15-Track and Field (95 M and 49 W)

**Fig. 9:**
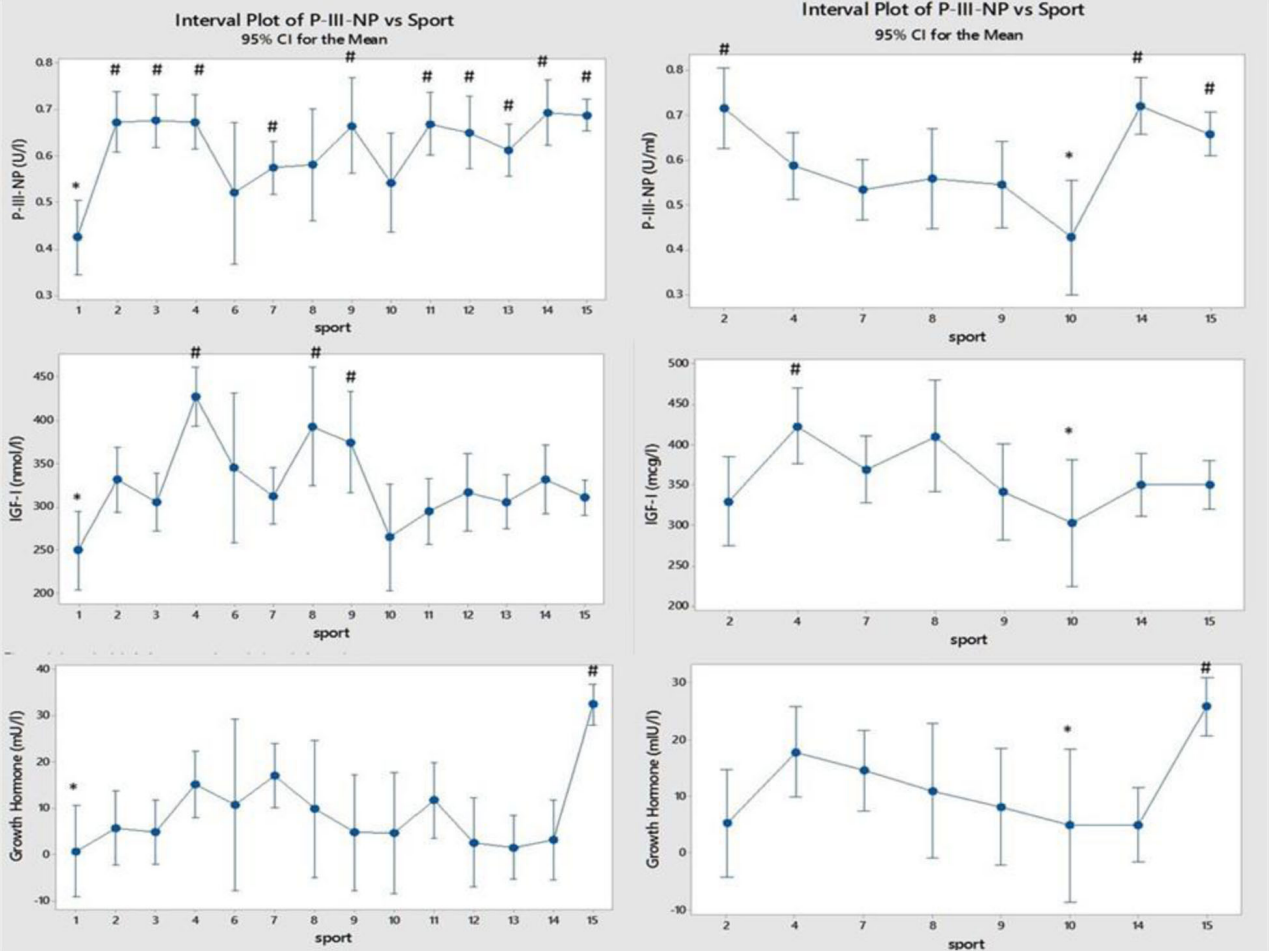
Comparison of Growth hormone, IGF-I and collagen marker P-III-NP between sports (men on the left and women on the right). Each sport is represented by a numerical code and M = men and W = women: 1-Power Lifting (18 M and 1 W), 2-Basketball (27 M and 14 W), 3-Football (Soccer; 37 M), 4 Swimming (100 M and 91 W), 5-Marathon (1 W), 6-Canoeing (7 M and 1 W), 7-Rowing (36 M and 25 W), 8-Cross Country Skiing (8 m and 9 W), 9-Alpine Skiing (11 M and 12 W), 10-Weight Lifting (10 M and 7 W), 11-Judo (26 M), 12-Bandy (19 M), 13-Ice Hockey (38 M), 14 Handball (23 M and 29 W) and 15-Track and Field (95 M and 49 W)

Using one-way analysis of variance the differences in means for each variable between sports and for men and women separately are illustrated in Figs. 2, 3, 4, 5, 6, 7, 8 and 9. Sports are represented by codes and the key to these is in the legend to each figure together with the number of volunteer elite athletes of each sex in each group.

Thus in Fig. 2, for men, weightlifters are older and shorter than in other sports while cross-country skiers are the youngest and lightest and basketball players the tallest. There are fewer volunteers and thus less data for women elite athletes but again basketball players are the tallest, weightlifters the shortest and cross-country skiers the lightest. Swimmers of both sexes were both young and relatively tall.

Figure 3 compares eLBM, eFM and BMI between the sporting groups. Cross-country skiers were not only lightest but also had the lowest (estimated) lean body mass, fat mass and BMI for both men and women. The differences in BMI between the groups closely match the differences in (estimated) fat mass. Surprisingly both LBM and eLBM were relatively small in Powerlifters where mean fat mass (FM) and eFM were large partly due to one outlier with a measured FM of 74 kg. In women there was a similar pattern in eLBM, eFM and BMI between sports.

Figure 4 shows LH, FSH and Testosterone between sports and between sexes. The testosterone concentrations in the powerlifters are on average remarkably small and 8 of the remaining sports had significantly larger values.

Figure 5 shows oestradiol, SHBG and Prolactin between sports and between sexes. All three were high in men from track and field sports where prolactin was also high in women. The ‘stress hormones’ cortisol, growth hormone and prolactin [4] were all high in both men and women from track and field.

Figure 6 provides the thyroid function test results between sports in men and women. The most notable feature is the low free T3 in male powerlifters and weightlifters and track and field athletes while free T4 was low in male canoeists. In women, TSH was high in track and field and free T4 low in cross-country skiers while swimmers and track and field athletes had significantly raised values.

Figure 7 shows the results for three IGF binding-proteins between sports. IGFBP-2 was high in male rowers and both men and women from track and field, while it was low in both alpine and cross-country skiing in men. IGFBP-3 was low in male powerlifters (but not weightlifters), canoers and rowers where it was also low in women. In both men and women the acid-labile subunit was low in basketball, weightlifting and track and field while it was relatively high in swimmers and rowers from both sexes.

Figure 8 shows the data on the bone marker osteocalcin and collagen markers ICTP and PICP. Most notably osteocalcin and ICTP are low in male powerlifters while osteocalcin was low in women from track and field.

Figure 9 shows data on growth hormone (GH) and the GH-sensitive markers IGF-I and P-III-NP. All three are relatively low in male powerlifters and weightlifters from both sexes. The GH-sensitive collagen marker P–III-NP and GH are relatively high in track and field in both sexes.

**Fig. 10:**
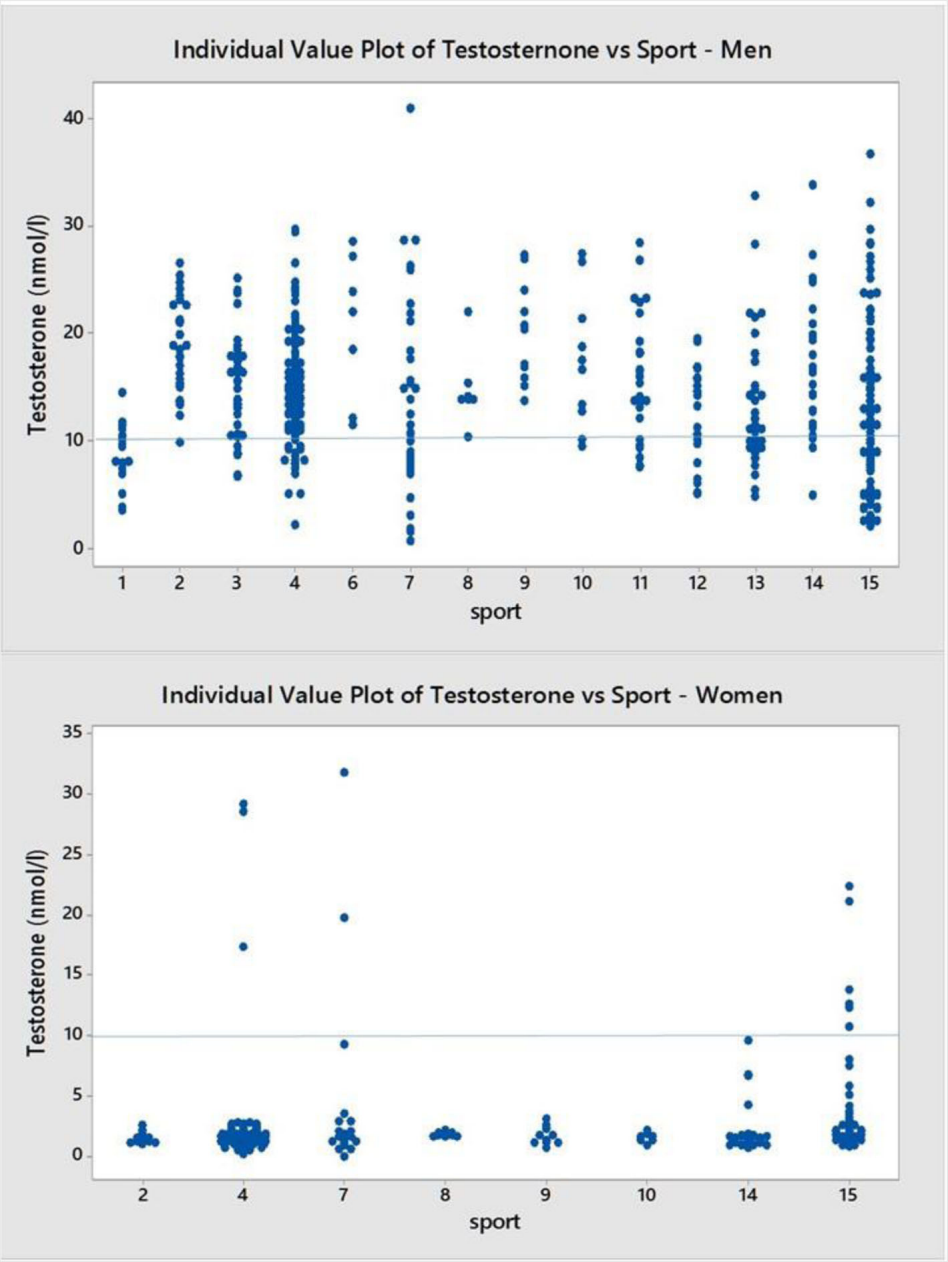
Above –Serum testosterone in 445 elite male athletes. The horizontal line is at 10 nmol/l. There were 113 (25.4%) men with a testosterone value less than 10 nmol/l. Below - Serum testosterone in 231 elite female athletes. Horizontal line is at 10 nmol/l. There were 11 of 231 (4.8%) athletes with testosterone level above 10 nmol/l; 3 of 88 swimmers, 2 of 25 rowers and 6 of 48 track and field athletes. Each sport is represented by a numerical code and M = men and W = women: 1-Power Lifting (18 M and 1 W), 2-Basketball (27 M and 14 W), 3-Football (Soccer; 37 M), 4 Swimming (100 M and 91 W), 5-Marathon (1 W), 6-Canoeing (7 M and 1 W), 7-Rowing (36 M and 25 W), 8-Cross Country Skiing (8 m and 9 W), 9-Alpine Skiing (11 M and 12 W), 10-Weight Lifting (10 M and 7 W), 11-Judo (26 M), 12-Bandy (19 M), 13-Ice Hockey (38 M), 14 Handball (23 M and 29 W) and 15-Track and Field (95 M and 49 W)

Figure 10 shows the individual testosterone levels in the different sports for male and female elite athletes. The horizontal line is set at 10 nmol/l which is the lower end of the reference range for non-elite men and the level set by the IAAF and IOC when setting up the ‘hyperandrogenism’ rule for female elite athletes [5, 6]. It shows a significant proportion of elite male athletes with a low concentration of testosterone (25.4%) and a smaller but significant proportion of women elite athletes with high values (4.8%).

Tables 1 and 2 contains the results of the binary logistic regression, showing which variables were significantly associated with a given sport either positively or negatively and the statistical level of this association and its direction (positive or negative). Thus, unsurprisingly basketball players were characteristically tall and powerlifters short.

**Table 1:**
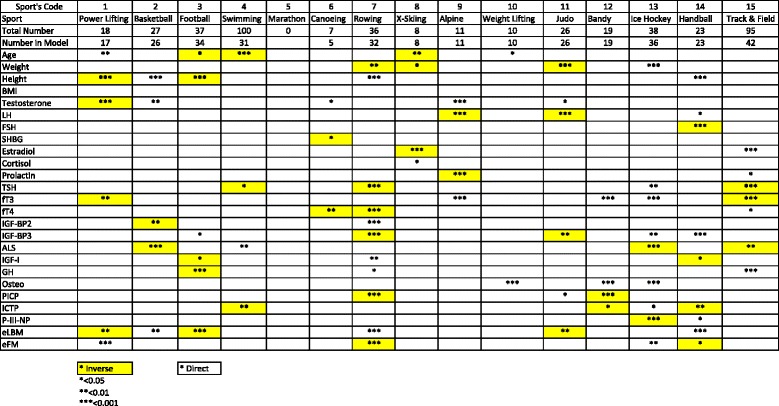
Significances of Logistic Regression of selective sports on body measurements (men)

**Table 2:**
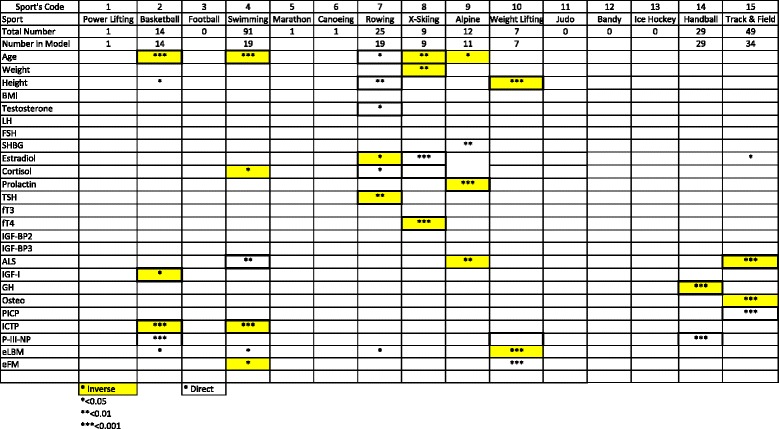
Significances of Logistic Regression of selective sports on body measurements (women)

Male powerlifters tended to be older while swimmers and cross-country skiers were younger than their colleagues in other sports. The data support the hypothesis that height was likely to be an advantage in basketball and may also be an advantage in rowing and handball but a disadvantage in powerlifting and football. Weight seemed a disadvantage in rowing but an advantage in ice-hockey. A higher testosterone was seen in basketball and alpine skiing while powerlifters had lower testosterone levels. BMI was not different in any group. LH was lower in alpine skiing and judo while FSH was lower in handball players. SHBG, like BMI and cortisol, showed no significant differences between sports. Oestradiol was lower in cross-country skiers and higher in track and field athletes. TSH was lower in rowers and track & field (athletes) and higher in ice hockey players. Free T3 was lower in powerlifters and track and field while it was higher in alpine skiers, bandy and ice hockey players. Free T4 was lower in canoeing and rowing.

IGFBP-2 was lower in basketball players and higher in rowers. IGFBP-3 was lower in rowers and judo players and higher in ice-hockey and handball players. ALS showed a different pattern being lower in basketball, Ice hockey and track and field while higher in swimmers. IGF-I showed a weak positive association with rowers while GH was lower in football players and higher in track and field.

Osteocalcin was higher in weight-lifters, bandy and ice hockey players while PICP was characteristically lower in rowers and bandy players. The other collagen markers ICTP was lower in swimmers and handball players and P-III-NP was lower in ice-hockey players.

Estimated body composition showed lean body mass to be lower in power-lifters and in football and judo players. LBM was higher in basketball players, rowers and handball players. Fat mass was lower in rowers but relatively high in power-lifters and ice-hockey players.

In women, there are fewer athletes and fewer significant findings. Basketball players, swimmers and cross-country skiers were characteristically younger than other sports while as with men, cross-country skiers were lighter than other sports. Like men, rowers were taller while unlike with men but as might be expected weightlifters were shorter. Again BMI showed no discriminating tendency, neither did testosterone, LH, FSH, cortisol, fT3, IGFBP-2 or IGF-I.

SHBG was higher in alpine skiers while oestradiol was higher in cross-country skiers. Prolactin was lower in alpine skiers, TSH lower in rowers and fT4 lower in cross-country skiers. ALS was higher in swimmers while it was lower in alpine skiers and track and field athletes. Growth hormone was lower in handball players, osteocalcin lower in track and field athletes who characteristically had higher levels of PICP. Basketball players and swimmers had lower levels of ICTP while P-III-NP was higher basketball and handball players.

Estimated lean body mass was surprisingly lower in weight-lifters who had a higher estimated fat mass.

## Discussion

This study has shown clear body composition and hormone concentration differences between athletes of different sporting disciplines of both sexes. These differences may contribute to the differences in *milieu interior* needed to excel in a given sport. We have used two complementary statistical methods to explore further the data. Firstly, analysis of variance has examined the magnitude of the differences between the body composition and endocrine profiles of the 15 Olympic sports that have been tested and is an extension of the analysis reported first in Healy et al. 2014 [1]. Secondly, in order to explore further the profile of body composition and hormone milieu for a given sport, we have used binary logistic regression to determine which of the measured variables appear ‘characteristic’ of a given sport. This has been done by comparing the profile of each sport against a pool of all the other sports and performed separately for men and women.

The results from the binary logistic regression may be compared with the differences in mean values of the variables shown in the figures. On most, but not all, occasions the results show a similar pattern; for example in men, there is a significantly higher age in powerlifters and weightlifters and younger age in swimmers and cross-country skiers. There are also several examples where there is little or no match. As might be expected female weight lifters were characteristically short but this was not the case with men.

For practical reasons it was only possible to measure body composition in 6 of the 15 sports in men and three in women. This was done at the time of data and blood sample collection post-event with a bioimpedance device of proven reliability and easy and fast to use in field studies such as this [7]. Bioimpedance analysis is to a degree dependent on hydration and ideally conditions of measurement should be standardised so far as hydration is concerned; this was not possible in this study and the results should be interpreted with this knowledge. Likewise the extreme ranges of body composition seen are beyond those used in validation of the method. The key factors in determining body composition are height, weight and sex [8]. In order to examine the effects of body composition across the whole group of 15 sports we analysed the available measured data and established a regression model from which we calculated an ‘estimated’ lean body mass (eLBM) and by subtracting this from the measured mass (M), and estimated fat mass (eFM). From Fig. 1 it can be seen that although we are comparing the model with the data from which it was derived, there is a very good fit between this model and the measured data for LBM and a less good but reasonable fit for FM.

Most surprisingly eLBM was low (and eFM high) in male powerlifters and women weightlifters. This may be true or possibly an artefact due to the bioimpedance method being unreliable in people with extreme variations in body composition. Apart from these observations body composition (in terms of muscle and fat) seemed little different between sports in women according to the logistic model although cross-country skiers (of both sexes) had the lowest eLBM, eFM and BMI by ANOVA. In men, basketball and handball players and rowers had the highest eLBM by both models. The differences in BMI between sports closely matched the differences in eFM in both sexes.

In both models testosterone concentrations were surprisingly low in powerlifters but not weightlifters, while large testosterone concentrations were a feature of basketball players and alpine skiers in the logistic model in men. There were no significant differences between sports in women for testosterone, LH or FSH. In the logistic model a low LH featured in male alpine skiers (where an association with a high testosterone was seen) and judo athletes while FSH was low in male handball players.

In men a low oestradiol was a feature of cross-country skiers in the logistic model while in contrast a high oestradiol was seen in women cross-country skiers. In men the highest average levels were in handball and track and field. In women a high SHBG was a feature of alpine skiers but SHBG did not feature in men. In both men and women a low prolactin characterised alpine skiers.

In the logistic model in both men and women, a low TSH was a distinguishing feature of rowers but mean values were equally low in swimmers, alpine skiers and track and field athletes while high values characterised male ice hockey players in the both models. Women from track and field had the highest mean TSH concentration but this was not a feature of the logistic model. Low average fT3 was seen in male power and weight-lifters and track and field athletes but was only important in power-lifters in the logistic model where a high fT3 distinguished alpine skiers and bandy and ice-hockey players; fT3 appeared of little significance in women. In men fT4 was low in both models for canoeing and rowing while for women it was low in both models for cross-country skiers.

In men, in the logistic model IGF-BP2 is low in basketball players and high in rowers, similar to the mean values where in addition IGF-BP2 was low in both cross-country and alpine skiers. In women although there are a few significant differences by ANOVA between sports for IGF-BP2 and -BP3 there are no differences between sports by binary logistic regression. In the logistic model in men however, IGF-BP3 is low in rowing and judo and high in ice hockey and handball. These results differ considerably from the means where power lifting is the lowest and rowing and judo not noticeably low, nor ice hockey and handball noticeably high. In both men and women there are peaks in the mean concentrations of IGF-BP3 and ALS in swimming and handball, this concordance between the sexes is not seen in the logistic model where IGF-BP3 is high in handball but not in swimmers while ALS is high in swimmers but not in handball. In men the logistic model shows ALS to be low in basketball, ice-hockey and track and field which matches the lows in ANOVA but here there is also the lowest mean in weight-lifters. It is noticeable that although IGF-BP3 and ALS are both GH-sensitive their patterns between sports in both mean values and in the logistic model are not always similar. This is also true for the other known GH-sensitive endocrine markers where in many instances the logistic model bears little likeness to the mean values from ANOVA.

Osteocalcin mean concentration is very low in power-lifters but this does not appear of any significance in the logistic model where low values characterise track and field where mean values are substantially higher than in power-lifters. In men weight-lifting, bandy and ice-hockey are characterised by high values that do not distinguish themselves in the ANOVA model. In women both models are in agreement over the low osteocalcin values in athletes from track and field. There is some discord between the models and the sexes for PICP, according to the logistic model a low value in men predicts rowers and bandy players while a high predicts track and field; by ANOVA although bandy players have a low mean PICP concentration there is no trough for rowers or peak for track and field. In women a high PICP is seen in track and field in both models.

One of the biggest discordances between models is for ICTP where low values are seen in the logistic model in men for swimming and handball but only in power-lifting by ANOVA while in women a low value strongly predicts basketball and swimming in the logistic model but there are no significant differences in ICTP concentrations between sports. There is no agreement between the models in men for P-III-NP where low values are observed in ice-hockey players (by logistic model) which is not reflected by ANOVA as the mean value is amongst the highest in ice hockey and a very low value seen in power-lifters. In women however, there is concordance between the models with high values in basketball and handball.

IGF-I levels are not associated with membership of any sporting group in women but high levels are associated with male rowers in the logistic model; rowers also have the highest mean level of IGF-I in both sexes. Low levels of growth hormone predict football in the logistic model in men and handball in women these results being discordant with the results from ANOVA where high values are seen in track and field in both men and women.

In power-lifting in men where mean GH levels are lowest is concordant with the low levels of GH-sensitive BP3, osteocalcin, ICTP, P-III-NP, and IGF-I. In each of these cases although the marker has a strong age-dependence and the power-lifters are the oldest, the differences between sports remains significant after adjusting for age (by regression analysis – data not shown).

In the first publication arising from this GH-2000 project the differences between marker levels between sports was attributed to differences in ages [3] but more detailed analysis has shown that although age plays an important part there are factors relating to the sport that prevail even when adjusting for age in all but two cases [1]. When allowing for age by regression analysis the differences between sports remain significant except for IGF-BP2 and BP3 in women [1] .

It is difficult to compare these results with other published data as there are little comparable data available. Rickenlund et al. [9] examined endocrine profiles in a group of female university athletes and matched controls but this paper focussed on hyperandrogenicity and menstrual status and not differences between sports. In our case the ‘hyperandrogenic’ (as defined by IAAF and IOC as having testosterone above 10 nmol/l [5, 6]) women were in swimming, rowing as well as track and field sports; the overall prevalence was 11/231 (4.8%, Fig. 10). This is highly relevant to the comparison of our data and those of Bermon et al. [10] who reported limited endocrine profiles in 849 elite female track and field athletes taking part in the 2011 IAAF World Championship in Daegu (South Korea). They removed the data from 5 women ‘suspected’ of doping and 5 ‘later diagnosed with hyperandrogenic 46 XY disorder of sex development (DSD)’ before analysis. They observed significant differences in testosterone, DHEAS and SHBG but not free testosterone between sports with ‘Throwers, sprinters and to a lesser extent jumpers having higher levels of androgenic hormones than long distance runners’. This may be simply because long distance runners are prone to develop a form of functional hypogonadism and thus have low testosterone levels [11]. They calculated the prevalence of ‘this type of medical condition’ (hyperandrogenism) as 7.1 per 1000. The prevalence of ‘hyperandrogenism’ in our smaller group of randomly selected elite female athletes from 8 Olympic sports is nearly seven times greater. The high prevalence of hyperandrogenic disorders of sex development (46,XY DSD) in sport has been explained by genes for stature that occur on the Y-chromosome rather than the high testosterone levels [12], which in normal people have only a small influence on the development of lean body mass [2].

Looking at the converse and accepting the IOC and IAAF value of 10 nmol/l as being the lower limit of the ‘normal’ range for serum testosterone in men, ‘hypoandrogenism’ was present in 113 of 445 (25.4%) elite male athletes from 15 Olympic sports. Since these blood samples were taken within 2 h of completing their event in a National or International competition there can be no doubt that these were elite athletes competing at the highest level. The low testosterone may be related to the gruelling training that elite athletes have to maintain as it has been shown that exercise to exhaustion in young troops can lead to a state of ‘functional hypogonadism’ that resolves spontaneously with rest and a good night’s sleep [13]. On the other hand it is possible that in some cases the low testosterone with normal LH and FSH may indicate the use of anabolic steroids that had been discontinued some time before the competition [14]. This example shows that simple correlations involving hormone levels and events does not necessarily indicate causation but may simply be a consequence of that event. Acute exercise itself has little effect on testosterone levels and so in the case of the data presented here, the timing of the blood sampling is unlikely to affect the number of low values [15]. There were no low values in basketball, canoeing, cross-country and alpine skiing and weight lifting but all other sports had a significant number of competing athletes with ‘hypoandrogenism’. Low free testosterone levels (0.23 nmol/l) were found by Bermon and Garnier in 101 of 795 (12.7%) elite male track and field athletes in their sample from the World athletics championship in Daegu [10]; they did not report the data for total testosterone. Thus it is clear that a very low testosterone level does not prevent an elite male athlete from competing in top events. In addition Bermon and Garnier [16] found no correlation between serum testosterone and performance in either men or women.

Cardinale and Stone [17] demonstrated higher testosterone levels in female sprinters than volleyball players as well higher testosterone levels in male sprinters than soccer and handball players. They also showed that both male and female sprinters managed a higher ‘countermovement jump (CMJ)’ than the volleyball players. By correlating the results between these two groups they showed an apparent correlation between endogenous testosterone and performance. They did not consider that this was more likely a ‘false correlation’ as they were effectively drawing a line between two distinct groups and the use of correlation in this situation is inappropriate [18], they should have looked at the relationship between CMJ and testosterone levels by adjusting for sports discipline; this was a serious methodological limitation. More recently Eklund et al. [19] related serum androgen levels to performance in a group of female Olympic athletes. They found no differences in testosterone levels between sports and no correlation between serum testosterone and performance but were able to show weak correlations between some androgen precursors and performance. In a recent review Bermon reported that he was able to find a relationship between free testosterone levels and performance such that those with the highest free testosterone levels had between 1.8 and 4.5% advantage [20]; they found no relation between free testosterone and performance in the elite men. In the full paper [16] it is clear that they showed no relationship between endogenous testosterone concentration and performance in elite women athletes nor, unlike the men, any differences in free testosterone between sports. Thus neither group showed a significant correlation between serum total testosterone (the endocrine variable used in the ‘hyperandrogenism’ rule) and performance. They did claim to show a relationship between (estimated) free testosterone and performance in five out of 21 sub-groups but in nine of these sub-groups, those with the lower testosterone performed better.

### Strengths and weaknesses

Our main strength is that we have examined comprehensive endocrine and anthropomorphic profiles in a large cohort of elite athletes of both sexes from a wide range of Olympic sports who were competing at national or international events. The blood samples were taken using a standardised protocol within two hours of completing their event, the serum separated promptly and stored at −80 °C until analysed. Analyses of serum samples were made by experts familiar with the methods and we have analysed the data extensively.

Our main weakness is that this was a supplementary study to the main GH-2000 project using remaining serum aliquots and consequently not all variables were measured on all athletes, thus the data set is incomplete. We did not know where volunteer athletes finished in their events and so we cannot match hormone profile to performance. We cannot exclude the possibility that any athlete was ‘doping’ although we consider that the fact that they volunteered to participate in a research study and signed a consent form that specifically excluded anyone who was currently or had previously used performance enhancing drugs made this unlikely together with the fact that there were no suspicious results to suggest this as a possibility. As the blood samples were taken within two hours of completing their event, they could have been taken at any time of day and thus they represent a random sample and may not be a true representative of the daily secretion. Likewise, the degree of hydration was not standardised although athletes had access to water and other drinks. This obligatory timing of sampling of the athletes was mandated by the agreement with the sporting authorities. It also meant that the state of hydration of each athlete at the time of body composition measurement was not standardised. The wide ranges of body composition in the volunteers is also outside the ranges of normal where the machines have been validated; measures of body composition must therefore be understood as measurements taken ‘in the field’. Likewise, the estimated body composition data can only be considered approximate.

## Conclusion

We have shown that, just as there are anthropomorphic differences between elite athletes from different events, there are also different hormonal profiles. We have not been able to elucidate whether this is the reason why athletes choose their events or whether this is a consequence of having trained and competed in their selected event over many years. There are clearly certain physical attributes that encourage individuals to pursue particular events (e.g. height and basketball) but also other events where they can modify their body to suit the event (e.g. weight control and cross-country skiing); it is possible that certain endocrine profiles favour success in a particular sport. We have shown that this is an area fertile for further research; an important ‘next step’ would be to examine endocrine profiles in relation to performance within events.

## Additional file

Additional file 1: Endocrine Profile Data from GH-2000 Project. (XLS 400 kb)

## Abbreviations

ALS: Acid-labile subunit
ANOVA: Analysis of variance
DSD: Disorder of sex development
eFM: Estimated fat mass
eLBM: Estimated lean body mass
FM: Fat mass
FSH: Follicle stimulating hormone
fT3: Free tri-iodothyronine
fT4: Free thyroxine
GH: Growth hormone
GH-2000: GH-2000 A Methodology for the Detection of Doping with Growth Hormone & Related Substances. EU Contract Number: BMH4 CT950678 Official Contractual period: 1st January 1996-31st January 1999
IAAF: International Association of Athletics Federations
ICTP: Carboxy-terminal cross-linked telopeptide of type I collagen
IGFBP-3: IGF Binding protein #3
IGFBP-2: IGF binding protein #2
IGF-I: Insulin-like growth factor 1
IOC: International Olympic Committee
LBM: Lean body mass
LH: Luteinising hormone
M: Mass (weight)
PICP: Carboxy-terminal propeptide of type I collagen
P-III-NP: Pro-collagen type III N-terminal peptide
SHBG: Sex hormone binding globulin
TSH: Thyroid stimulating hormone
